# A New Hotspot of Cave Leptodirini (Coleoptera: Leiodidae) from the Romanian Carpathians †

**DOI:** 10.3390/insects16080806

**Published:** 2025-08-04

**Authors:** Cristian Sitar, Marius Kenesz, Lucian Barbu-Tudoran, Oana Teodora Moldovan

**Affiliations:** 1 Zoological Museum, Babeș Bolyai University, Clinicilor 5, 400006 Cluj-Napoca, Romania; 2 Cluj-Napoca Department, Emil Racovita Institute of Speleology, Clinicilor 5, 400006 Cluj-Napoca, Romania; 3 Electron Microscopy Center “Prof. C. Craciun”, Faculty of Biology & Geology, “Babes-Bolyai” University, Clinicilor 5-7, 400006 Cluj-Napoca, Romania; 4 Electron Microscopy Integrated Laboratory, National Institute for R&D of Isotopic and Molecular Technologies, Donath 67-103, 400293 Cluj-Napoca, Romania

**Keywords:** subterranean, beetles, *Protopholeuon*, new taxa, Metaliferi Mountains

## Abstract

Recently explored caves in the Apuseni Mountains (Romanian Carpathians) are inhabited by cave Coleoptera belonging to the subterranean genus *Protopholeuon* (Leiodidae: Cholevinae: Leptodirini). In this study we undertake a detailed revision of the genus *Protopholeuon* (Csiki, 1904), endemic to Metaliferi Mountains (Apuseni Mountains). Until now, this genus has been considered monospecific, with *Protopholeuon hungaricum* being its only species. We redescribe *P. hungaricum* with enhanced morphometric and morphological details and add the description of a new subgenus, *Pachyphallus* n. subgen., and four new species: *P.* (s. str.) *rusescuae* n. sp., *P. (Pachyphallus) ponoricum* n. sp., *P. (Pachyphallus) grohotae* n. sp., *P. (Pachyphallus) hodobanae* n. sp. All these species are endemic to one or two neighbouring caves. Morphological descriptions, including the male abdominal sternite IX, male and female genitalia, revealed distinct characteristics for the new taxa. Scanning electron microscopy (SEM) was employed to describe ultrastructural details. The geographical distribution of *Protopholeuon* in the Metaliferi Mountains, along with natural barriers represented by impermeable rocks, further supports the separation of these new taxa. This comprehensive study not only enriches the taxonomy of the Leptodirini tribe but also highlights the rich and underestimated biodiversity of the Carpathian Mountains’ cave habitats.

## Simple Summary

This study reveals previously undocumented diversity within *Protopholeuon*, a genus of cave beetles endemic to the Metaliferi Mountains in the Romanian Carpathians. Through detailed morphological and ultrastructural analysis, we redescribe the known species and describe one new subgenus and four new species, each of which is restricted to one or two caves. These findings highlight the exceptional and localised biodiversity in karst systems and emphasise the urgent need for targeted conservation measures to protect these unique subterranean habitats.

## 1. Introduction

Romania’s subterranean habitats (including caves and other superficial subterranean environments) have more than 300 troglobionts according to Dryad, https://doi.org/10.5061/dryad.9ghx3fff9 [1,2], terrestrial taxa adapted to life in subterranean environments. These adaptations generally include a lack of eyes, an elongated body, legs, and antennae, as well as depigmentation. Of these species, approximately 80% are endemic to a single cave or up to three caves [1], due to subterranean geological barriers, as soluble rocks are not continuous, and to strict adaptation features that make surface migration difficult.

The best-represented group of subterranean fauna in Romania and Europe in general are the beetles (Coleoptera), with the second most diverse beetle group represented by the Leiodidae, especially its Leptodirini tribe (Cholevinae) [3,4]. To date, approximately 1000 species and more than 235 genera are recognised [5,6,7,8]. Most species of Leptodirini were found in the Iberian Peninsula, France, the Mediterranean islands, the Southern Alps, the Italian and Balkan peninsulas, the Carpathian Mountains, southern Russia, the Caucasus, the Middle East, and Iran [4].

The Carpathians constitute a significant biodiversity hotspot in Europe for many fauna groups [9,10,11], especially the Western Carpathians of Romania (Apuseni Mountains) [12]. In Romania, the Leptodirini tribe is represented by 8 genera and 49 endemic species [2,13]. The endemic cave beetle genera inhabiting the Romanian Carpathians are *Mehadiella* J. Frivaldszky 1880, *Banatiola* Decu 1967, *Sophrochaeta* Reitter 1884, *Closania* Jeannel, 1928, and *Tismanella* Jeannel 1928, distributed in the Southern Carpathians, and *Drimeotus* L. Miller 1856, *Pholeuon* C. Hampe 1856, and *Protopholeuon* Jeannel 1923, inhabiting the Western unit (the Apuseni Mountains).

**Figure 1:**
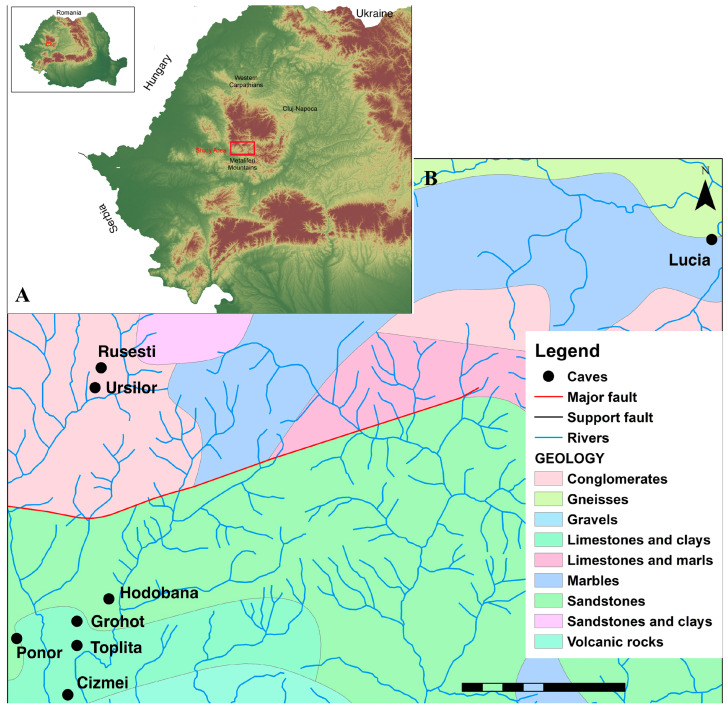
The position of the Metaliferi Mountains in northwestern Romania ((**A**) the red square), modified after ESA [26]. (**B**) The geology of the area where the populations of *Protopholeuon* were identified with different types of limestones and other non-soluble rocks, modified after Ianovici and Giușcă [27] and Enciu [28]; the red line represents a prominent fault in the area.

The Leptodirini genera of the Apuseni Mountains have endemic subgenera for each of the mountain units, with *Pholeuon* (s. str.) Hampe 1856 and *Drimeotus* (*Bihorites*) Jeannel 1923 in the Bihor Mountains, *Drimeotus* (s. str.) Miller 1856 and *Pholeuon* (*Parapholeuon*) Ganglbauer 1887 in the Pădurea Craiului Mountains, and *Protopholeuon* present only in caves of the Metaliferi Mountains (Figure 1). *Pholeuon* and *Drimeotus* were intensively collected and revised by Racoviță [14,15,16,17,18] and Moldovan [3,19,20,21,22], and molecular phylogeny and phylogeography were undertaken by Bucur [13] and Năstase-Bucur et al. [23].

The genus *Protopholeuon*, with the type species *Protopholeuon hungaricum*, was described by Csiki [24] from Lucia Cave, at the northern border of the Metaliferi Mountains. The description was incomplete, and later Jeannel [25] published a more accurate morphological description of this genus, including its male genitalia. Over the last few decades, more attempts have been made to protect the Romanian caves, with one of the objectives being to inventory the cave fauna in lesser-explored areas of the Carpathians. The Metaliferi Mountains were such an area, and newly discovered caves were biospeleologically explored. Several *Protopholeuon* populations were identified as new taxa by using morphometric, morphological, and fine structural features. All the taxa belonging to *Protopholeuon* are low-range endemics, and conservation measures are also proposed for these rare taxa.

## 2. Materials and Methods

### 2.1. Study Area

The Metaliferi Mountains represent the structural unit of the southern Apuseni Mountains, which stretches between the Arieș River to the north and the Mureș River to the south (Figure 1A) [27]. From a geological point of view, this structural unit has four subunits that are stratigraphically characterised by Mesozoic ophiolites (rock masses embedded in flysch deposits), Jurassic and Cretaceous limestones, and Cretaceous and Neogene eruptive flysch. The limestone blocks of the Metaliferi Mountains appear as individualised outcrops, which do not continue laterally under the flysch and range in size from a few meters to several kilometres long. This structure can also be defined by geological limits represented by flysch or various types of limestone (Figure 1B), as well as faults resulting from tectonic activity. These are the possible geological barriers that separate basins and cave invertebrate populations even in relatively small areas.

### 2.2. Sampling

All the beetle specimens were collected by hand and no traps or baits were used to avoid over-collection. They were collected from the Lucia, Grohot, Izbucul Topliței, Ponor, Rusești, Hodobana, Cizmei, and Urșilor de la Bulzești caves (Figure 1, Table 1) and preserved in 95% Ethanol. For comparisons, *Pholeuon* (s. str.) *knirschi intermittens* (Knirsch, 1913) was collected from Huda Laptelui de Piatră Cave (Bihor Mountains; Table 1), the closest available species of *Pholeuon* in the area.

### 2.3. Morphological Analyses and Measurements

**Figure 2:**
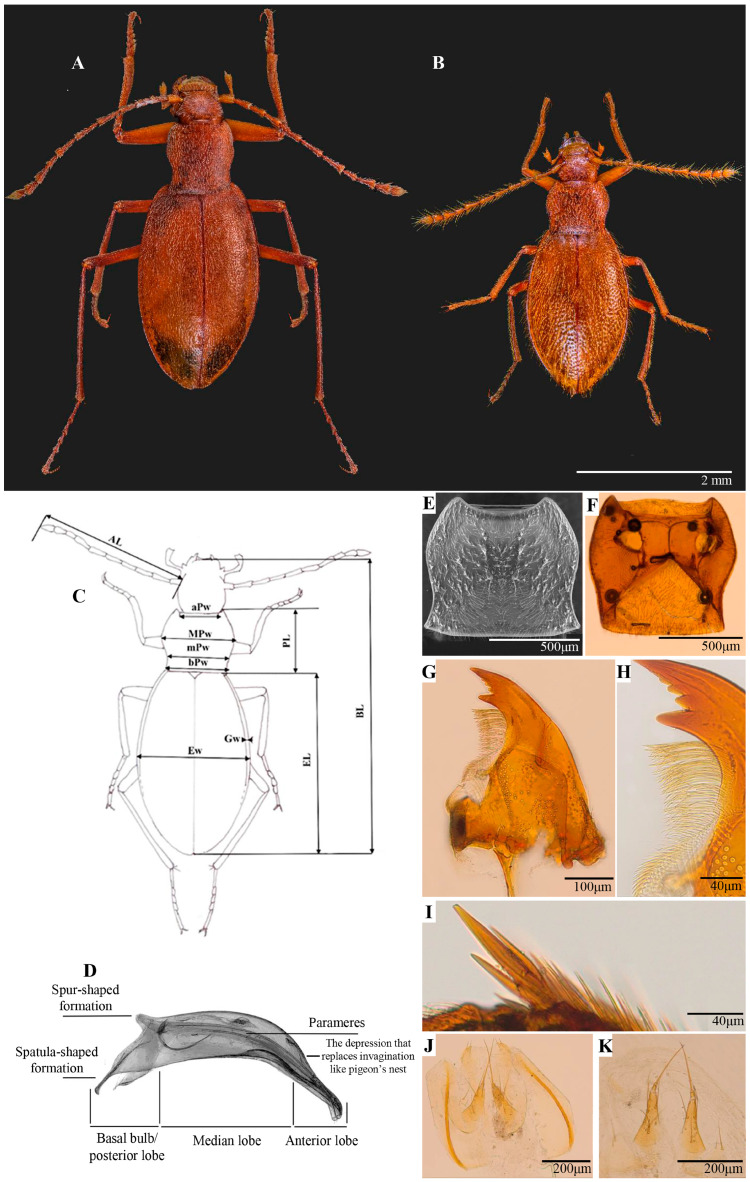
Habitus and separate structures of Leptodirini: (**A**) habitus of *Pholeuon* (s. str.) *knirschi intermittens*; (**B**–**K**): (**B**) habitus of *Protopholeuon* (s. str.) *hungaricum*, (**C**) used measurements, (**D**) used terms for aedeagus (lateral view), (**E**) pronotum (dorsal view), (**F**) prothorax (ventral view), (**G**) mandible (dorsal view), (**H**) mandibular apex bearing ramified hairs along inner edge, (**I**) polydentate spurs on metatibiae, (**J**) female genitalia, (**K**) setae on gonostyli and coxites.

The genital structures were removed from the bodies and then fixed on microscope slides in glycerol. The exact process was also used for other body parts that were subjected to ultrastructural analysis. Afterwards, the beetles were dry mounted on cardboard and examined under a stereomicroscope (Olympus SZX16, Tokyo, Japan) and for detailed examination under an Olympus BX51 microscope (Tokyo, Japan). All drawings were prepared using a camera lucida mounted on both microscopes. Measurements were made using a micrometre. The nomenclature and all taxonomically important morphological characters were studied for comparison, as described by Fresneda et al. [8], Perreau [29], and Ćurčić et al. [30,31,32], and for the fine structures by Moldovan et al. [33]. Characters analysed for comparison are presented in Figure 2C, and the measurements are in Table 2.

### 2.4. Scanning Electron Microscopy (SEM)

The specimens were also prepared for scanning electron microscopy (SEM) using the turbomolecular pumped coater Quorum Q150T ES (Quorum Technologies, Laughton, UK) from the Integrated Electron Microscopy Laboratory (LIME) of the National Institute for Research and Development of Isotopic and Molecular Technologies (INCDTIM), Cluj-Napoca, Romania, and examined with an SEM Hitachi SU8230 (Hitachi Group, Tokyo, Japan) (LIME-INCDTIM, Cluj-Napoca, Romania). The nomenclature and all taxonomically important morphological characters were studied for comparison, following [8,34,35,36].

## 3. Results

### 3.1. Redescription of the Subgenus *Protopholeuon* with the Type Species *P. hungaricum*

Taxonomy: Family Leiodidae Fleming, 1821

     Subfamily Cholevinae Kirby, 1837

     Tribe Leptodirini Lacordaire, 1854

     Genus *Protopholeuon* Jeannel, 1923

     Subgenus *Protopholeuon* Jeannel, 1923

     Type species *P. hungaricum* (Csiki, 1904)

The description of *Protopholeuon hungaricum* provided by Jeannel [25] lacks details on the male genitalia (aedeagus and endophallus) and female genitalia, especially the spermatheca, which is taxonomically important for Leptodirini. We added ultrastructural details. This species was compared to *Pholeuon* (s. str.) *knirschi intermittens* (Knirsch, 1913), the closest taxon to *Protopholeuon* from phylogenetic [23] and geographic points of view.

Holotype and paratypes were not designated in the original descriptions by Csiki [24] and Jeannel [25]. The material studied by Jeannel was not found in the Department of Cluj-Napoca, Emil Racovita Institute of Speleology Collection, where it had been previously deposited. Fresh specimens were collected from Lucia Cave, the type locality.

*Syntypes*: 45♂♂, 100♀♀, Lucia Cave, Poieni karstic basin, Metaliferi Mountains, Romania, legit. Geza Rajka, 4 March 2001 (published by Bucur [23]); 4♂♂, 6♀♀ legit. C.S. and M.K., 17 September 2022. Material is deposited in the ISER Cluj-Napoca Entomological Collection.

*Diagnosis:* Medium-sized Leptodirini, with body and parts smaller than *Pholeuon* (s. str.) *knirschi intermittens* (Figure 2A). Compared to *P.* (s. str) *knirschi intermittens*, *P.* (s. str.) *hungaricum* has pholeuonoid body shape, elliptical outline, and slightly narrower anteriorly (Figure 2B, Table 2). Aedeagus medium size, elongated, slightly curved, weakly sclerotised compared to *Pholeuon* (s. str.) *knirschi intermittens* aedeagus.

*Body*: Elliptically shaped, reddish-brown. Mean total body length 3.65 mm ♂ and 3.82 mm ♀ (Table 2). Integument with less evident microsculpture, more pronounced with reticulated appearances in head posterior region. Body covered with long, yellowish pubescence, erect on head and recumbent on pronotum and elytra, like in *Pholeuon* (s. str.) *knirschi intermittens*.

*Elytra punctuation*: More evident than in *Pholeuon* (s. str.) *knirschi intermittens* (Figure 2A,B). In dorsal view, punctuation less evident around scutellum and elytral disc. Distance between punctures gradually increases from elytral disc towards lateral edges and posterior region. Posteriorly, punctures larger in surface and shallower in depth.

*Head:* Longer than wider (HL/HW; Table 2), non-retractable, without eyes, with a strongly expressed occipital carina. Clypeus, strongly delineated, showing several long hairs and one pore on each side. Labium apex, in dorsal view, deeply bilobed, with three long setae on each lobe and tufts of short sensory setae and labial palps in a depression between the two lobes.

**Figure 3:**
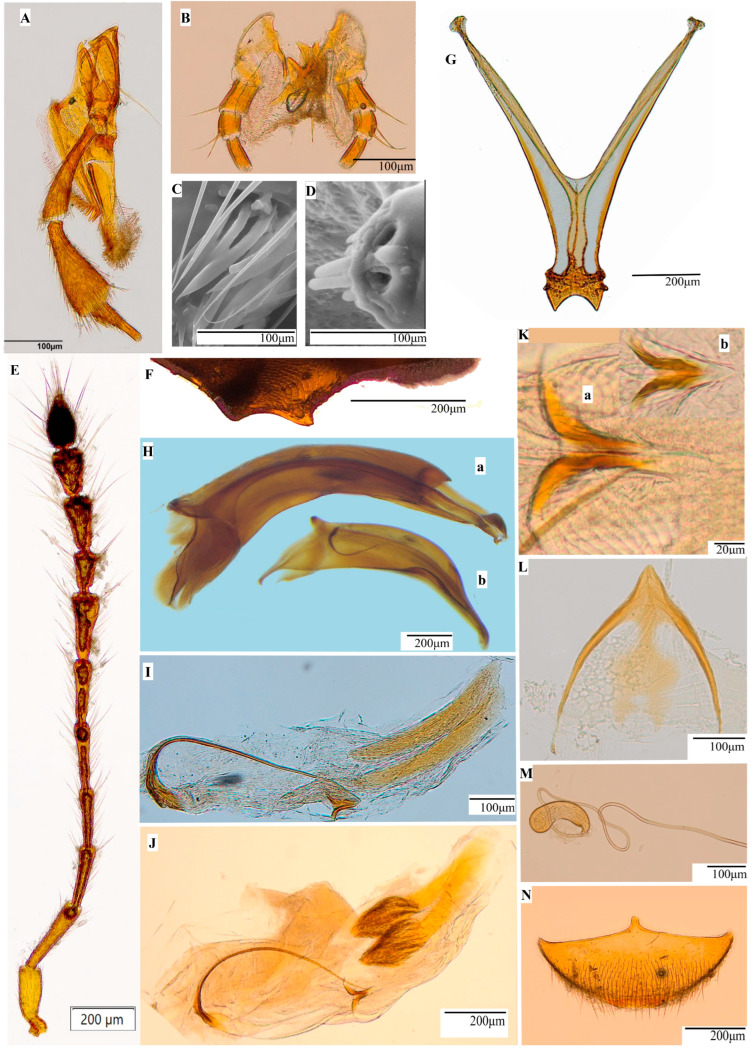
*Protopholeuon* (s. str.) *hungaricum*: (**A**) maxilla with palpomeres and galea; (**B**) labium; (**C**) elongated basiconic sensilla at the base of 3rd palpomere; (**D**) campaniform sensilla on the apical region of palpomere; (**E**) antennae; (**F**) mesosternal carina; (**G**) metendosternite; (**H**) Aedeagus (lateral view) of *Pholeuon* (s. str.) *knirschi intermittens* (**a**) and *P.* (s. str.) *hungaricum* (**b**); (**I**) endophallus in *P.* (s. str.) *hungaricum*; *(***J**) endophallus in *P.* (s. str.) *knirschi intermittens*; (**K**) U-shaped sclerotised pieces in *P.* (s. str.) *knirschi intermittens* (**a**) and *P.* (s. str.) *hungaricum* (**b**); male urite IX (**L**), spermatheca (**M**) and female uroventrite VIII (**N**).

*Mouthparts:* Maxillary palpomeres (Figure 3A) thin and elongated; first palpomere thin at base and widens towards articulation with second palpomere; second palpomere widened apically; third palpomere shorter than palpomeres 1 and 2, gradually thinning towards apex. Palpomere 3 with basiconic sensilla in apical region, palpomere 2 elongated and few short ones. Apex of palpomere 3 surrounded by one digitiform sensilla, round shaped. Below digitiform sensilla scattered campaniform sensilla (Figure 3D). Base of palpomere 3 with numerous elongated basiconic sensilla arranged in bundles on outer side of maxillary palp (Figure 3C).

Galea flattened and covered with dense pubescence like curved hairbrush; lacinea shorter than galea and covered with thick, curved hairs (Figure 3A). Labium concave anteriorly and covered with numerous short hairs (Figure 3B). Inside concavity, two long setae. Labium sides with labial palps, segmented, each with three palpomeres; first palpomere with one long seta apically oriented outwards; palpomere 2 much shorter than palpomere 1 and slightly thinner. Close to palpomere 1, two long setae, oriented outwards in articulation area with nodules; longest palpomere 3 with thickness cca—½ of palpomere 1; apical region with numerous basiconic sensilla.

Wide mandible base, with curved apex having two teeth and two weakly contoured denticles between them (Figure 2G); masticatory edge with ramified hairs that appear to merge at base (Figure 2H); outer curvature with beveled area and numerous short setae and one very long; apically, outer curvature, depression and prominent seta, much shorter than previous.

*Antennae:* 2.57 mm ♂ and 2.42 mm ♀, base thin and thickened tip (Figure 3E). Antennomeres integument resembles scales. Antennomeres I-VI slightly widened apically; base of antennomere I with five chaetic sensilla, possible mechanoreceptors for antenna position. Antennomeres VII, IX, and X strongly widened apically and narrower than first six antennomeres; antennomere VIII short, elongated, moderately widened apically, about half as long as antennomere VII; antennomere VIII with elongated sensilla chaetica around articulation with antennomere IX; antennomere XI pear-shaped, with numerous mechanoreceptors and chemoreceptors, with thicker base than antennomere X, and apically pointed. In *Pholeuon* (s. str.) *knirschi intermittens*, antennomere IX thick base as antennomere X with an ovoid shape, elongated and thinning slightly towards apex.

*Pronotum:* Like in *Pholeuon* (s. str.) *knirschi intermittens*, small, short bell-shaped (PL = 0.87 mm ♂, 0.89 mm ♀) (Figure 2E,F); wider in anterior third, significantly wider than head (mean MPw = 0.93 mm ♂, 0.98 mm ♀); edges well rounded anteriorly, somewhat concave posteriorly (mean mPw = 0.78 mm ♂, 0.83 mm ♀), subparallel base. Pronotal base straight, slightly wider than elytral base (bPw = 0.80 mm ♂, 0.85 mm ♀); anterior margin slightly convex medially (aPw = 0.63 mm ♂, 0.66 mm ♀); angles small, rounded, obtuse; posterior angles sharp, prominent, slightly projected backwards; disc convex.

*Elytra:* Elliptic, elongated (EL = 2.58 mm ♂, 2.73 mm ♀). Significantly wider than pronotum. Ratio between mean width and length 1:1.79 ♂ and 1:1.75 ♀. Maximum width near middle (Ew = 1.44 mm ♂, 1.56 mm ♀). Lateral margins, slightly arched, forming a narrow marginal groove, regular in shape and not widened in middle area (Gw = 0.8 mm ♂, ♀). Disc convex, steeply declining apically in lateral view. Scutellum large, subtriangular. Apex attenuated, rounded. Pygidium covered by elytra.

*Legs:* Elongated and slender. Femora broadened basally, gradually tapering towards apex. Protibia thinner compared to mesotibia and metatibia. Mesotibia bears few fine spines, whereas metatibiae bear multiple long and thick spines. Forelegs metatibiae have polydentate spurs (Figure 2I). First tarsomere twice as long as wide. Each tarsomere bears two long setae distally. Tarsal claws long, slender, apically curved, and sharply pointed.

*Mesoventral keel:* Poorly represented, carrying one sharp tooth. Metaventrite anterior border (convex) with few setae (Figure 3F).

*Metatergal apparatus:* Long metascutum wings, narrowing from middle towards apex. Alacrista apophysis elongated, with narrow edges. Apophysis tip bears three to five setae.

*Metendosternite:* Furcal arms long, slightly more than one and a half times trunk length. Apical area as sharp arc. Terminal apophysis reduced but visible. Posterior lamina narrow, not reaching apex, but broader. Furcal trunk slightly longer than wide. Median keel diverges posteriorly (Figure 3G).

*Aedeagus:* Comprises three regions: posterior (basal), median, and anterior lobes (Figure 2D and Figure 3(Hb)). Posterior lobe slightly elongated and rounded in dorsal view. In lateral view, where parameres attach, rounded, with curved and oriented posteriorly spur-shaped structure. Posterior lobe basal blade resembles one elongated spatula, uniformly sclerotised, forming one end hook. Spatula oriented posteriorly. Median lobe described by Fresneda et al. [8]. Anterior lobe tapers anteriorly, with dorsoventrally flattened apex extending beyond parameres. Dorsal invagination, resembling pigeon’s nest in *Pholeuon* (s. str.) *knirschi intermittens*, absent (Figure 3(Hb)); in lateral view, replaced by depression, giving sinuous appearance between median and anterior lobes. Dorsally, apex rounded with shallow central depression. In lateral view, strongly curved downward, like eagle’s beak. Apical area sides with rows of fine sensilla chaetica, interspersed with delicate nodosities.

*Endophallus:* Elongated, tubular, and membranous aspect (Figure 3I). Stylet long and thin with basal plate Y-shaped, slightly sclerotised, better developed than in *Pholeuon* (Figure 3J). Stylet curved, tapering from base to tip. Apical reinforcing bands weakly sclerotised. Two groups of spines present at *Pholeuon* are missing in *Protopholeuon* (Figure 3I). Middle area, between stylet apical part and reinforcement bands base with small U-shaped sclerotised piece; U-shaped piece with sharp, elongated arms curving outward. Base hyaline (transparent), while arms sclerotised (Figure 3K).

*Parameres:* Elongated, thin, almost straight dorsally and weakly arched laterally, gradually narrowing distally, each with rounded, widened apex, club-like. Each paramere with three equidistant long pointed setae in subapical position and pores like small depressions.

*Male urite IX:* Well developed, subtriangular, with pointed apex and inner cup-shaped projection; oval-shaped gap on internal projection (Figure 3L).

*Female genitalia:* Ovipositor weakly sclerotised, with elongated coxites, slim with sclerotised inner margins and slightly curved; wide, rounded apex with two weakly sclerotised zones, each with two long and very short setae. Gonostyli elongated, thin, gradually narrowing distally, pointed apically, straight; each stylus with one apical seta and four other setae arranged laterally; two setae, one long and one short, at gonostyli base. 

*Spermathecal complex:* Short, membranous, strongly curved (Figure 3M). Apical lobe rounded and wide, and basal lobe slightly elongated. Basal lobe with recess on internal curvature connected with one membranous accessory gland; it continues with very long, tubular spermiduct.

*Female uroventrite VIII:* Wide, with dense pilosity on posterior half. Anterior border with anteriorly developed spiculum, narrow base and rounded apex (Figure 3N). Spiculum sides straight and curved anteriorly.

### 3.2. *Protopholeuon (s. str.) rusescuae* Sitar & Moldovan sp. nov.

urn:lsid:zoobank.org:act:9456EC63-FB81-440D-892D-3A51D69AD083

*Etymology*: Named after the cave in which it was discovered.

*Examined material:* Holotype 1 ♂, Rusești Cave, Bulzești–Rusești karstic basin, Metaliferi Mountains, Romania (Table 1), legit. O.T.M. and M.K., November 2018. Deposited in the ISER Cluj-Napoca Entomological Collection.

Paratypes 2♀♀, 7♂♂ Rusești Cave, Bulzești–Rusești karstic basin, Metaliferi Mountains, Romania (Table 1), legit. O.T.M. and M.K., November 2018; 4♀♀, 6♂♂ legit. C.S., M.K., and Ruxandra Bucur, 25 October 2021; 2 ♀♀ Urșilor Cave, Bulzești–Rusești karstic basin, Metaliferi Mountains, Romania, legit. O.T.M. and M.K., November 2018.

*Metatergal apparatus:* Like in *P.* (s. str.) *hungaricum*.

*Metendosternite:* Furcal arms and trunk like in *P.* (s. str.) *hungaricum.* Apical area like sharp arc. Terminal apophysis reduced but still present. Posterior lamina arrow not reaching apex, but slightly wider and median keel slightly more divergent posteriorly than in *P.* (s. str.) *hungaricum*.

**Figure 4:**
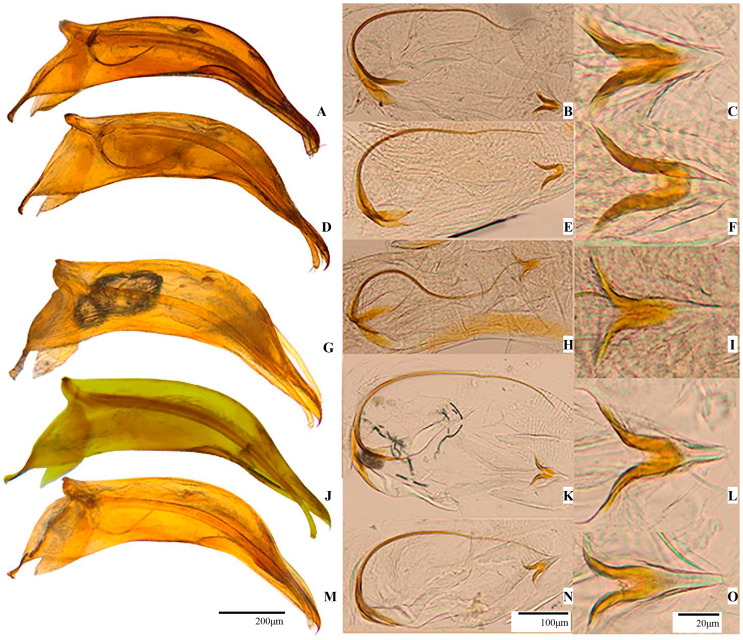
Aedeagus, endophallus, and U-shaped piece of *Protopholeuon* (s. str.) *hungaricum* (**A**,**B**,**C**), *P.* (s. str.) *rusescuae* Sitar & Moldovan sp. nov. (**D**,**E**,**F**), *P. (Pachyphallus) ponoricum* Sitar & Moldovan sp. nov. (**G**,**H**,**I**), *P. (P.) grohotae* Sitar & Moldovan sp. nov. (**J**,**K**,**L**), *P. (P.) hodobanae* Sitar & Moldovan sp. nov. (**M**,**N**,**O**).

*Aedeagus:* Like in *P.* (s. str.) *hungaricum* in overall shape, with some distinct differences (Figure 4D). General curvature slightly more pronounced, and apex more elongated and narrower. Posterior lobe slightly more expanded, rounded in dorsal view. Viewed laterally, posterior lobe shows one rounded spur-shaped formation as in *P.* (s. str.) *hungaricum* where parameres are attached. Basal blade shaped like one elongated spatula, tapering towards end with anteriorly oriented hook. Spatula shorter compared to other species described in this study.

Median lobe margins almost parallel in dorsal view and slightly curved in lateral view. Anterior lobe like in *P.* (s. str.) *hungaricum*. Dorsally, each side of apical areas with rows of fine sensilla chaetica.

*Endophallus* (Figure 4E): Stylet longer but Y-shaped piece with arms like in *P.* (s. str.) *hungaricum*. U-shaped piece with thicker arms and more robust appearance (Figure 4F). Sclerotised region pronounced, and arms curvature moderate. Hyaline base still visible but slightly less prominent than in *P.* (s. str.) *hungaricum*.

*Parameres:* Elongated, thin, almost straight dorsally and weakly arched laterally, gradually narrowing distally, with apex widened club-like. Each paramere with three equidistant, long pointed setae in subapical position and pores in small depressions.

**Figure 5:**
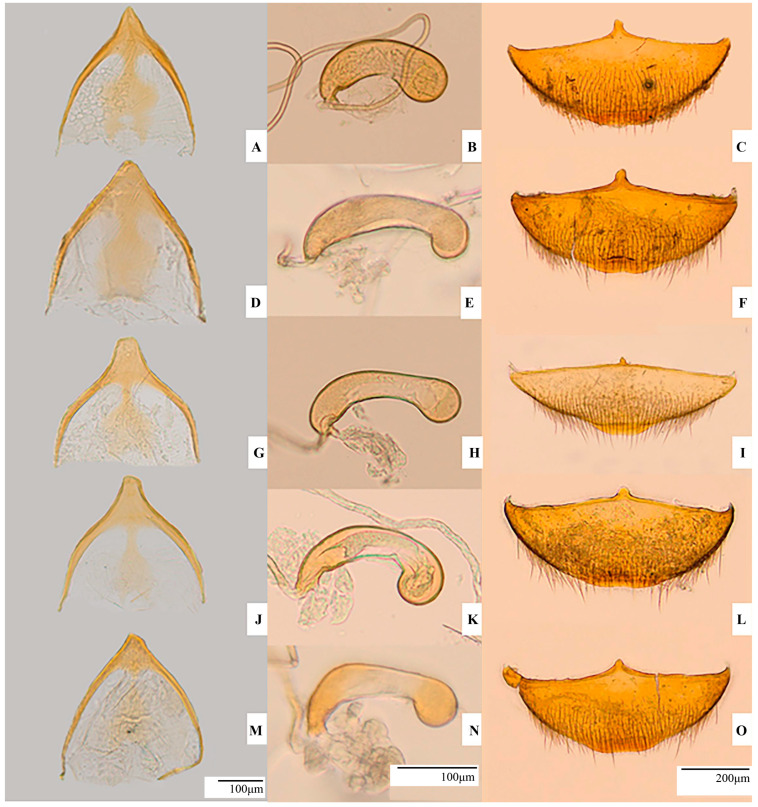
Male urite IX, spermatheca and female uroventrite VIII of *Protopholeuon* (s. str.) *hungaricum* (**A**,**B**,**C**), *P.* (s. str.) *rusescuae* Sitar & Moldovan sp. nov. (**D**,**E**,**F**), *P. (Pachyphallus) ponoricum* Sitar & Moldovan sp. nov. (**G**,**H**,**I**), *P. (P.) grohotae* Sitar & Moldovan sp. nov. (**J**,**K**,**L**), *P. (P.) hodobanae* Sitar & Moldovan sp. nov. (**M**,**N**,**O**).

*Male urite IX*: Well developed, with arched arms, generally bell-shaped, and rounded, dome-shaped apex. Inner cup-shaped prominence like in *P.* (s. str.) *hungaricum* (Figure 5D).

*Spermathecal complex:* Short, more elongated than in *P.* (s. str.) *hungaricum* (Figure 5E). Anterior part slightly arched ventrally. Club-like anterior part smaller than in other species described here.

*Female uroventrite VIII:* Wide as in *P.* (s. str.) *hungaricum* with anteriorly developed, elongated spiculum but slightly thicker and better defined (Figure 5F).

### 3.3. Description of the Subgenus *Protopholeuon (Pachyphallus)* Sitar & Moldovan subgen. nov.

urn:lsid:zoobank.org:act:1264462C-7151-4FBC-A1A5-F8B92254ABC6

*Taxonomy:* Family Leiodidae Fleming, 1821

   Subfamily Cholevinae Kirby, 1837

   Tribe Leptodirini Lacordaire, 1854

   Genus *Protopholeuon* Jeannel, 1923

   Subgenus *Pachyphallus* subgen. nov.

   Type species *P. (Pachyphallus) ponoricum*

*Etymology: Pachyphallus* (in Greek pachýs = thick and fallós = phallus) describes the male’s aedeagus, which is thicker and bulkier in the posterior half, compared to *Protopholeuon* (s. str.).

**Figure 6:**
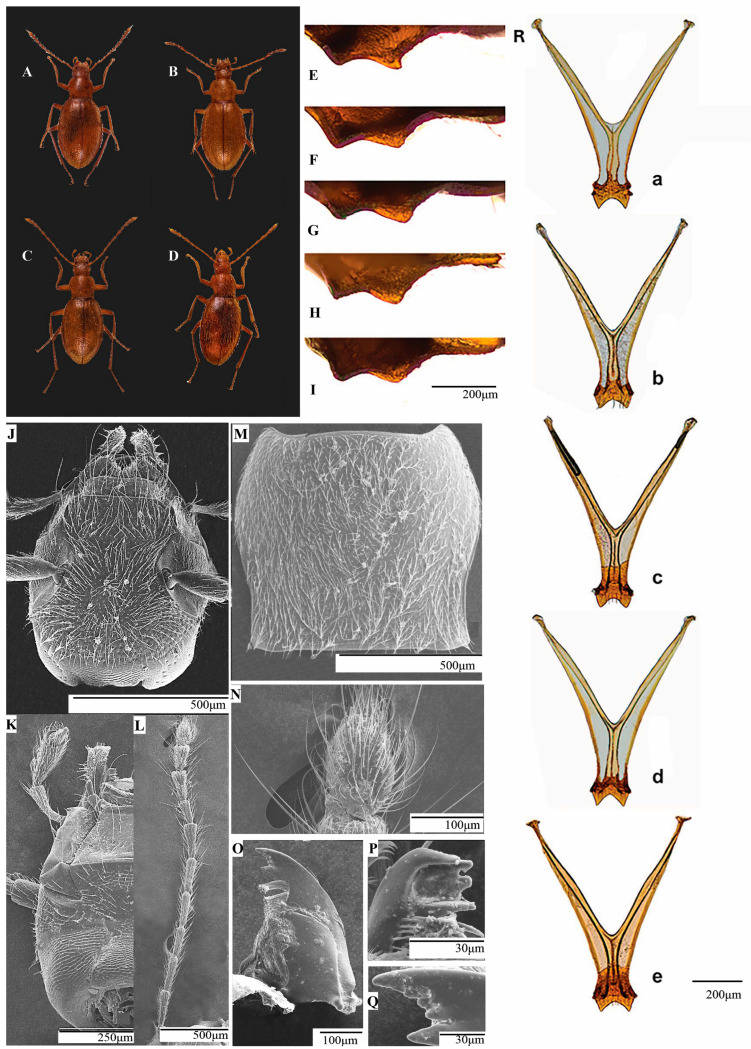
Habitus and mesosternal carina in *Protopholeuon* (s. str) *rusescuae* Sitar & Moldovan sp. nov. (**A**,**F**), *P. (Pachyphallus) ponoricum* Sitar & Moldovan sp. nov. (**B**,**G**), *P. (P.) grohotae* Sitar & Moldovan sp. nov. (**C**,**H**), and *P. (P.) hodobanae* Sitar & Moldovan sp. nov. (**D**,**I**). Mesosternal carina (for comparison) in *Protopholeuon* (s. str) *hungaricum* (**E**). Structural and ultra-structural details in *P. (Pachyphallus) ponoricum*: (**J**) head in dorsal view; (**K**) head in ventral view; (**L**) antennae; (**M**) pronotum in dorsal view; (**N**) antennomere XI with a pear shape; (**O**) mandible; (**P**) lacinea; (**Q**) curved mandible apex with two teeth and two weakly contoured denticles between them. (**R**) Metendosternite in *P.* (s. str.) *hungaricum* (**a**) *P. (P.) rusescuae* (**b**) *P. (P.) ponoricum* (**c**) *P. (P.) grohotae* (**d**) *P. (P.) hodobanae* (**e**).

*Diagnosis:* Body shape pholeuonoid, elliptical, and slightly narrower anteriorly, similar in size to *Protopholeuon* (s. str.) (Table 2). Elytra elliptical and widened posteriorly. Colour reddish brown (Figure 6B). Morphological differences from *Protopholeuon* s. str. in aedeagus, endophallus, male urite IX, spermathecal complex, and female uroventrite VIII.

*Aedeagus:* More robust and slightly wider with more pronounced basal part; in lateral view, more pronounced curvature; apex rounder. Posterior lobe more prominent where parameres attach than in *Protopholeuon* (s. str.); oriented posteriorly and apically with one small hook.

We describe three new species of P. (*Pachyphallus*) subgen. nov., *P. (P.) ponoricum* sp. nov., *P. (P.) grohotae* sp. nov., *P. (P.) hodobanae* sp. nov. Morphological differences between these new species were in aedeagus, endophallus, male urite IX, spermathecal complex, and female uroventrite VIII, all described in detail below and illustrated in Figure 4G–O and Figure 5G–O. The three species have no significant morphological differences in the other body parts. Dimensions of head, pronotum, elytra, antennae, and total length of the body for all three species are presented in Table 2.

### 3.4. Protopholeuon (Pachyphallus) ponoricum Sitar & Moldovan sp. nov.

urn:lsid:zoobank.org:act:D672507D-83CB-488C-BC38-24CE5C203F6E

*Holotype:* 1♂, Ponor Cave, Rișculiței karstic basin, Metaliferi Mountains (Table 1), Romania, legit. O.T.M. and M.K., November 2018. Deposited in the ISER Cluj-Napoca Entomological Collection.

*Paratypes:* 6♀♀ Ponor Cave, Rișculiței karstic basin, Metaliferi Mountains (Table 1), Romania, legit. O.T.M. and M.K., November 2018; 1♂, 4♀♀ Ponor Cave, Rișculiței karstic basin, Metaliferi Mountains (Table 1), Romania, legit. C.S., M.K., and Ruxandra Bucur 25 October 2021. Deposited in the ISER Cluj-Napoca Entomological Collection.

*Body:* Mean total body length 3.98 mm ♂ and 4.00 mm ♀ (Table 2).

*Head:* Longer than wider (HL/HW: 1:1.18), non-retractable, without eyes (Figure 6J,K).

*Mouthparts:* Like in *P.* (s. str.) *hungaricum* (Figure 6O–Q).

*Antennae:* Like in *P.* (s. str.) *hungaricum* (Figure 6L,N).

*Pronotum*: Like in *P.* (s. str.) *hungaricum* (Figure 6M).

*Elytra:* Slightly rounder in shape than in *P.* (s. str.) *hungaricum* both distally and laterally. Mean width and length ratio 1:1.65 ♂ and 1:1.72 ♀.

*Legs:* Like in *P.* (s. str.) *hungaricum*.

*Metatergal apparatus:* Like in *P.* (s. str.) *hungaricum*.

*Metendosternite:* Furcal arms very long, stretched, slightly undulated, more than three times trunk length (Figure 6(Rc)); apical area like pointed arc. Posterior lamina strongly reduced. Median keel strongly divergent in middle part.

*Aedeagus* (Figure 4G)*:* Medium-sized, elongated, and slightly curved, weakly sclerotised. Posterior lobe typical of *P.* (*Pachyphallus)*. Median lobe nearly parallel in dorsal view, with upper half more curved than basal half and bearing large, prominent nodules. Parameres long, thin, slightly arched laterally, with rounded, club-like apices; each paramere bears three apical setae, one of which is longer than other two. Apex dorsoventrally flattened and longer than parameres.

*Endophallus:* Typical for *Protopholeuon* (Figure 4H). Anterior part with numerous formations on integument resembling rooster crest. Stylet curved, longer than in *Protopholeuon* (s. str.). Both stylets less sclerotised than in *Protopholeuon* (s. str.). U-shaped piece (Figure 4I) with thin and elongated arms; arms symmetrically curved, forming wide angle. Apex rounded and gently curved outward.

*Male urite IX:* Like in all *Protopholeuon*, but inner cup-shaped prominence less outlined than in *Protopholeuon* (s. str.). Surface of internal projection without gap (Figure 5G).

*Female genitalia:* Ovipositor weakly sclerotised, with elongated coxites; slim with inner margins sclerotised and slightly curved; wide, rounded apex with two weakly sclerotised zones with three setae each. Gonostyli elongate, thin, gradually narrowing distally, pointed apically. Each stylus with one apical seta and four other setae arranged laterally. Gonostyli base with two setae, one long and one short.

*Spermathecal complex:* Small, membranous, curved, with rounded apical part. Median area thinner than apical and basal parts. Basal part with a recess on internal curvature with connected membranous accessory gland. Spermatheca basal end thin continues with long, tubular spermiduct (Figure 5H).

*Female uroventrite VIII:* Narrow, with dense pilosity in basal half. Upper (transverse) border with short, small and round spiculum (Figure 5I).

### 3.5. Protopholeuon (Pachyphallus) grohotae Sitar & Moldovan sp. nov.

urn:lsid:zoobank.org:act:F5A8F2DF-692B-47E5-A336-4813C137965F

*Holotype:* 1♂, Grohot Cave, Grohot karstic basin, Metaliferi Mountains, Romania, legit. O.T.M. and M.K., November 2018. Deposited in the ISER Cluj-Napoca Entomological Collection.

*Paratypes:* 8♀♀, 10♂♂ Grohot Cave, Grohot karstic basin, Metaliferi Mountains, Romania (Table 1), legit. O.T.M. and M.K., November 2018; 10 ♀♀, 3♂♂ paratypes from Topliței Cave, Grohot karstic basin, Metaliferi Mountains, Romania, legit. C.S., M.K., and Ruxandra Bucur, October 2021. Deposited in the ISER Cluj-Napoca Entomological Collection.

*Metendosternite:* Like in *P. (P.) ponoricum* (Figure 6(Rd)).

*Aedeagus* (Figure 4J)*:* Comprising one posterior lobe with one rounded formation and one sharp spine oriented posteriorly; this structure smaller and less prominent than in *P. (P.) ponoricum*. Median lobe thinner than in *P. (P.) ponoricum*, with no nodules. Dorsally, apical area with rows of sensilla chaetica on both sides. Spatula short and oriented posteriorly. Apex dorsoventrally flattened and longer than parameres.

*Endophallus* (Figure 4K)*:* Stylet less sclerotised than in *P.* (s. str.) *hungaricum*, shorter and less arched than in *P. (P.) ponoricum*. U-shaped piece more compact, with shorter and more curved arms. Sclerotised regions slightly wider at centre, giving robust appearance. Hyaline base less distinct than in *P. (P.) ponoricum* (Figure 4L).

*Male urite IX:* Well developed, with arched arms, generally bell-shaped and prominent apical part developed anteriorly (Figure 5J). Protuberance narrower than in *P. (P.) ponoricum*, like truncated pyramid. Inner cup-shaped prominence less outlined than in *P. (P.) ponoricum.*

*Spermathecal complex* (Figure 5K)*:* Median area thinner than apical and basal parts; constricted appearance, elongated and strongly arched. Anterior part more prominent than in *P. (P.) ponoricum.*

*Female uroventrite VIII* (Figure 5L)*:* Wide, spiculum much larger than in *P. (P.) ponoricum*. Spiculum apex rounded.

### 3.6. Protopholeuon (Pachyphallus) hodobanae Sitar & Moldovan sp. nov.

urn:lsid:zoobank.org:act:1D4349AF-6CC9-4DD8-88ED-F226EE8CE84A

*Holotype:* 1♂, Hodobana Cave, Grohot karstic basin, Metaliferi Mountains (Table 1), Romania, legit. O.T.M. and M.K., November 2018. Deposited in the ISER Cluj-Napoca Entomological Collection.

*Paratypes:* 4♀♀, 7♂♂ Hodobana Cave, Grohot karstic basin, Metaliferi Mountains (Table 1), Romania, legit. O.T.M. and M.K., November 2018; 1♂, Cizmei Cave, Grohot karstic basin, Metaliferi Mountains, Romania, legit. O.T.M. and M.K., November 2018. Deposited in the ISER Cluj-Napoca Entomological Collection.

*Metendosternite:* Furcal arms very long, slightly undulated, extending more than three times trunk length (Figure 6(Re)). Apical area and terminal apophysis like in *P. (P.) ponoricum* and *P. (P.) grohotae*. Posterior lamina significantly reduced, nearly absent apically, base slightly more developed. Median keel like in *P. (P.) ponoricum* and *P. (P.) grohotae*.

*Aedeagus* (Figure 4M): Posterior lobe with rounded formation, more prominent than in *P. (P.) grohotae* but smaller than in *P. (P.) ponoricum*, slightly arched upward. One small apical hook present, resembling that in *P. (P.) ponoricum*. Median lobe thin and lacks nodules. Dorsally, apical area with rows of sensilla chaetica and clearly depressed apex, narrower and sharper than in other *P. (Pachyphallus)* species.

*Endophallus:* Stylet longer than in *P.* (s. str.) *hungaricum*, but shorter than in *P. (P.) ponoricum* and *P. (P.) grohotae* (Figure 4N). U-shaped piece elongated, with long, slender arms that curve outward significantly; arm tips long, sharply pointed, and slightly curved inward. Sclerotisation stronger, with visible hyaline base (Figure 4O).

*Male urite IX:* Well developed, with arched arms, generally bell-shaped. Apical prominence less developed than in *P. (P.) ponoricum* and *P. (P.) grohotae*. Inner cup-shaped prominence lesser outlined (Figure 5M).

*Spermathecal complex* (Figure 5N)*:* Spermatheca less arched and more elongated than in *P. (P.) grohotae* and *P.* (s. str.) *hungaricum*. Hump-like thickening dorsally and slightly arched ventrally.

*Female uroventrite VIII* (Figure 5O)*:* Narrower than in *P.* (s. str.) *hungaricum* and *P. (P.) grohotae*, but broader than in *P. (P.) ponoricum*. Spiculum short and narrower than in *P. (P.) grohotae*, more prominent than in *P. (P.) ponoricum*, with pointed apex.

### 3.7. Dichotomous key of *Protopholeuon* species

**1** Body robust (length: 4.82 mm ♂, 5.07 mm ♀) (Figure 2A). Elytra (length: 3.31 mm ♂, 3.53 mm ♀) with fine punctation and short hairs. Aedeagus median lobe with dorsal invagination like a pigeon’s nest (Figure 3(Ha)); stylet with Y-shaped basal plate weakly sclerotised; reinforcement bands bear at one end a group of sclerotised spines with club-like appearance (Figure 3J)..............................................*Pholeuon (s. str.) knirschi intermittens*

**-** Body small (length: 2.58 mm ♂, 2.73 mm ♀) (Figure 2B). Elytra (length: 2.58 mm ♂, 2.73 mm ♀) with evident punctation and long, dense hairs. Aedeagus median lobe without dorsal invagination like a pigeon’s nest (Figure 3(Hb)); stylet with Y-shaped basal plate slightly sclerotised; reinforcement bands lack spines (Figure 3I).........................................**2**

**2** Aedeagus thin. Spur-shaped projection, slightly curved anteriorly, where parameres are attached. Spatula oriented posteriorly, elongated, with one small hook. Endophallus U-shaped piece robust, with wider arms and uniform sclerotisation; arms thicker, without extreme curvature. Female uroventrite VIII upper (transverse) border with rounded apex of anteriorly developed spiculum. Male urite IX triangular with sharp tip prominence. Metendosternite with furcal arms slightly more than 1.5 of trunk length.............................................................................................................**3**

**-** Aedeagus thick. Posterior lobe rounded, well-contoured, and prominent. Spur-shaped projection oriented posteriorly, apically with one small hook oriented posteriorly. Spatula oriented ventrally and slightly arched. Endophallus U-shaped piece with long and close arms and elongated appearance. Female uroventrite VIII upper (transverse) border with sharp apex of anteriorly developed spiculum. Male urite IX apex prominence wide and truncated, resembling pyramidal trunk. Metendosternite with furcal arms three times trunk length.............................................................................................**4**

**3** ..................................................................*Protopholeuon* (s. str.) *hungaricum*

**-** Posterior lobe slightly more expanded, spatula shorter; endophallus U-shaped piece more robust, hyaline base less visible; uroventrite VIII with thicker spiculum...............................................*Protopholeuon* (s. str.) *rusescuae* Sitar & Moldovan sp. nov.

**4** Spatula short and oriented posteriorly. Endophallus U-shaped piece elongated, with long, slender arms that curve outward significantly; arms tips long, sharply pointed, and slightly curved inward. Sclerotisation stronger, hyaline base visible.................................*Protopholeuon (Pachyphallus) hodobanae* Sitar & Moldovan sp. nov.

**-** Spatula oriented ventrally. Endophallus U-shaped structure with thin and elongated arms; arms symmetrically curved, forming a wider angle, with rounded and gently curved outward tips. .... *Protopholeuon (Pachyphallus) ponoricum* Sitar & Moldovan sp. nov.

**-** Spatula short and oriented posteriorly. Endophallus U-shaped piece more compact, with shorter and more curved arms. Sclerotised areas slightly wider at centre, giving a robust appearance. Hyaline base less distinct .......................................................*Protopholeuon (Pachyphallus) grohotae* Sitar & Moldovan sp. nov.

## 4. Discussion

Separated by the Arieș River, *Protopholeuon* (Metaliferi Mountains) and *Pholeuon* (Bihor Mountains) genera share some characteristics, but morphologically essential features (especially at the aedeagus level) differ. These differences include the distinct general habitus, large and short pronotum, the elytra’s large punctuation, and the elytra’s narrow and regular marginal groove in *Protopholeuon*. The main differences of the *Protopholeuon* aedeagus are the absence of the pigeon-nest invagination characteristic of *Pholeuon*, the relatively smaller and less sclerotised aedeagus, and a much shorter median lobe.

The clypeus, labium, and other mouthparts, and the types of sensilla and their shape are described here for the first time in *Protopholeuon*. On the mandible, we observe a characteristic dentition, distinct from that reported by Jeannel [25]. Metatergal apparatus, metendosternite, male urite IX, female genitalia, the gonostyles and spermathecal complex, female uroventrite VIII, and mesoventral keel were also described here for the first time as specific features in differentiating the newly described subgenus *P.* (*Pachyphallus*). This new subgenus is morphologically like *Protopholeuon* s. str. with a clear differentiation based on the internal structures like the aedeagus, metendosternite, spermathecal complex, male urite IX, and female uroventrite VIII, where significant differences are observed. The most prominent difference in the aedeagus between the two subgenera is in the posterior lobe (basal bulb), where spur-shaped and spatula-shaped formations differ in shape, size, and orientation. The spermathecae in *P. (Pachyphallus)* are more elongated and less arched than in *Protopholeuon* s. str.

The geographical distribution of the cave populations of *Protopholeuon* in the Metaliferi subterranean environment, with possible natural barriers represented by the patchy limestone distribution intercalated with non-soluble rocks and the local hydrological network, supports the separation of subgenera and species based on the observed morphological differences.

The discovery of the new taxa also expands the distribution of *Protopholeuon* throughout the entire karstic area of the Metaliferi Mountains. The genus *Protopholeuon*, previously considered monospecific, has proven to comprise a complex of short-range endemic taxa, each confined to discrete karst systems within the Metaliferi Mountains. This taxonomic revision enhances our understanding of the diversity of subterranean beetles in the Romanian Carpathians. Given the high degree of endemism and the restricted distributions of these newly described species, future conservation actions should prioritise entire basins that may harbour more unique and vulnerable subterranean species.

The presence of Ponor, Cizmei, Toplița, Hodobana, and Grohot caves within the ROSPA0132 Metaliferi Mountains protected area places them under the jurisdiction of the Directive of the European Union (79/409/EC; Directive 2009/147/EC [37]), thus ensuring a certain level of legal protection. However, additional conservation measures are needed, including the expansion of the Natura 2000 network through the designation of a Site of Community Importance (SCI) to encompass caves from the Bulzești–Rusești and Poieni karst basins—specifically Urșilor, Rusești, and Lucia caves—and provide comprehensive protection for these ecologically significant habitats [38], especially facing the increased challenges of climate change [39] and anthropic pressure, like increased dryness and tourism in caves.

Our study highlights the need for the conservation of all karstic areas, including small and isolated caves, which can contain endemic fauna, not only from the Coleoptera group. The subterranean fauna has scientific importance for the study of low-range endemics in restrictive cave environments (characterised by a lack of light, saturated air relative humidity, and constant temperature), and it provides the perfect environment for studying adaptation and evolution processes.

## Figures and Tables

**Table 1:** Physical characteristics and location of the studied caves.

Cave	Air Temperature (°C)	Karstic Basin	Absolute Altitude (m)	Rock Type	Coordinates
Ponor	11.0	Rîșculiței	480	Limestone	46.236979° N, 22.733602° E
Hodobana	11.0	Grohot	470	Limestone	46.257245° N, 22.785063° E
Grohot	11.4	Grohot	600	Limestone	46.255324° N. 22.772105° E
Topliței	11.4	Grohot	530	Limestone	46.235599° N, 22.780594° E
Cizmei	11.5	Grohot	430	Limestone	46.221721° N, 22.769745° E
Urșilor	9.9	Bulzești–Rusești	620	Metamorphosed limestone	46.306961° N, 22.759897° E
Rusești	10.0	Bulzești–Rusești	650	Metamorphosed limestone	46.323134° N, 22.776788° E
Lucia	9.6	Poieni	570	Metamorphosed limestone	46.361234° N, 23.006931° E
Laptelui de Piatră	9.3	Arieșul Mare	960	Limestone	46.468274° N, 22.926859° E

**Table 2:** Mean values (mm) of the morphological measurements of the main characteristics in *Pholeuon* (s. str.) *knirschi intermittens* (from Racoviță [17]) and *Protopholeuon* representatives (standard deviations in italics); see also (Figure 2C): n = number of individuals, F = females, M = males, BL = body length (head was measured in normal position); PL = pronotum length; aPw = apical width of pronotum; MPw = maximum width of pronotum; mPw = minimum width of pronotum; bPw = width of pronotum at base; EL = length of elytra; Ew = width of elytra; Gw = groove width; AL = length of antennae.

	Species	*Pholeuon* (s. str.) *knirschi intermittens*	*Protopholeuon (s. str.) hungaricum*	*Protopholeuon* (s. str.) *rusescuae sp. nov.*	*Protopholeuon (Pachyphallus) ponoricum sp. nov*	*Protopholeuon (Pachyphallus) grohotae sp. nov.*	*Protopholeuon (Pachyphallus) hodobanae sp. nov.*
Females	n	42	100	6	10	18	6
	BL	5.64 ± *0.10*	3.82 ± *0.03*	4.16 ± *0.16*	4.00 ± *0.10*	3.93 ± *0.14*	4.12 ± *0.25*
	PL	1.31 ± *0.03*	0.89 ± *0.01*	0.86 ± *0.03*	0.81 ± *0.03*	0.79 ± *0.03*	0.82 ± *0.02*
	aPw	0.88 ± *0.03*	0.66 ± *0.01*	0.64 ± *0.02*	0.64 ± *0.01*	0.62 ± *0.01*	0.66 ± *0.03*
	MPw	1.43 ± *0.04*	0.98 ± *0.01*	1.01 ± *0.03*	9.43 ± *0.01*	9.44 ± *0.03*	9.78 ± *0.04*
	mPw	1.26 ± *0.05*	0.83 ± *0.01*	0.85 ± *0.03*	0.81 ± *0.02*	0.81 ± *0.02*	0.82 ± *0.04*
	bPw	1.26 ± *0.04*	0.85 ± *0.01*	0.84 ± *0.04*	0.82 ± *0.02*	0.80 ± *0.02*	0.84 ± *0.04*
	EL	3.83 ± *0.10*	2.73 ± *0.02*	2.53 ± *0.09*	2.49 ± *0.05*	2.46 ± *0.07*	2.61 ± *0.16*
	Ew	2.30 ± *0.06*	1.56 ± *0.01*	1.60 ± *0.04*	1.50 ± *0.04*	1.52 ± *0.05*	1.54 ± *0.06*
	jw	-	-	0.08 ± *0.01*	0.08 ± *0.01*	0.08 ± *0.01*	0.07 ± *0.01*
	AL	3.48 ± *0.12*	2.42 ± *0.03*	2.40 ± *0.10*	2.31 ± *0.08*	2.26 ± *0.07*	2.27 ± *0.10*
	BL/AL	0.61 ± *0.02*	0.63	0.57	0.57	0.57	0.55
Males	n	34	100	14	8	14	7
	BL	5.20 ± *0.14*	3.65 ± *0.04*	3.89 ± *0.18*	3.98 ± *0.04*	4.07 ± *0.13*	3.74 ± *0.12*
	PL	1.28 ± *0.04*	0.87 ± *0.01*	0.83 ± *0.04*	0.83 ± *0.04*	0.83 ± *0.03*	0.80 ± *0.02*
	aPw	0.84 ± *0.02*	0.63 ± *0.01*	0.61 ± *0.02*	0.64 ± *0.06*	0.64 ± *0.02*	0.62 ± *0.01*
	MPw	1.24 ± *0.04*	0.93 ± *0.01*	0.94 ± *0.03*	9.21 ± *0.07*	1.00 ± *0.04*	0.92 ± *0.02*
	mPw	1.17 ± *0.05*	0.78 ± *0.01*	0.78 ± *0.03*	0.78 ± *0.07*	0.85 ± *0.03*	0.77 ± *0.02*
	bPw	1.18 ± *0.04*	0.80 ± *0.01*	0.78 ± *0.04*	8.14 ± *0.07*	0.84 ± *0.03*	0.79 ± *0.01*
	EL	3.61 ± *0.10*	2.58 ± *0.03*	2.39 ± *0.12*	2.48 ± *0.04*	2.56 ± *0.04*	2.37 ± *0.06*
	Ew	2.17 ± *0.06*	1.44 ± *0.01*	1.45 ± *0.06*	1.44 ± *0.01*	1.56 ± *0.06*	1.44 ± *0.04*
	jw	-	-	0.07 ± *0.01*	0.07 ± *0.01*	0.08 ± *0.01*	0.08 ± *0.01*
	AL	3.78 ± *0.14*	2.57 ± *0.04*	2.45 ± *0.10*	2.60 ± *0.05*	2.38 ± *0.06*	2.39 ± *0.05*
	BL/AL	0.72 ± *0.03*	0.7	0.63	0.65	0.58	0.63

## Data Availability

The original contributions presented in this study are included in the article. Further inquiries can be directed to the corresponding author.
