# Correction: Sitar et al. A New Hotspot of Cave Leptodirini (Coleoptera: Leiodidae) from the Romanian Carpathians. *Insects* 2025, *16*, 806

**DOI:** 10.3390/insects16121251

**Published:** 2025-12-10

**Authors:** Cristian Sitar, Marius Kenesz, Lucian Barbu-Tudoran, Oana Teodora Moldovan

**Affiliations:** 1 Zoological Museum, Babeș-Bolyai University, Clinicilor 5-7, 400006 Cluj-Napoca, Romania; 2 Cluj-Napoca Department, Emil Racovita Institute of Speleology, Clinicilor 5, 400006 Cluj-Napoca, Romania; 3 Electron Microscopy Center “Prof. C. Craciun”, Faculty of Biology & Geology, Babeș-Bolyai University, Clinicilor 5-7, 400006 Cluj-Napoca, Romania; lucian.barbu@ubbcluj.ro; 4 Electron Microscopy Integrated Laboratory, National Institute for R&D of Isotopic and Molecular Technologies, Donath 67-103, 400293 Cluj-Napoca, Romania

## Text Correction

Because the name *Pachyphallus* [1] is preoccupied we propose replacing it with *Pachyandrus*. Therefore, all mentions of *Pachyphallus* throughout the article need to be revised and replaced with *Pachyandrus*.

Etymology: *Pachyphallus* (in Greek pachýs = thick and andros = man) describes the male’s aedeagus, which is thicker and bulkier in the posterior half, compared to *Protopholeuon* (s. str.).

The authors state that the scientific conclusions are unaffected. This correction was approved by the Academic Editor. The original publication has also been updated.

## References

[1] Sitar C., Kenesz M., Barbu-Tudoran L., Moldovan O.T. (2025). A New Hotspot of Cave Leptodirini (Coleoptera: Leiodidae) from the Romanian Carpathians. Insects.

